# Multiplexed immune profiling and 3D co-culture assays to assess the individual checkpoint therapy response in head and neck squamous cell carcinoma

**DOI:** 10.3389/fonc.2025.1622008

**Published:** 2025-08-08

**Authors:** Verena Schweihofer, Daniela Schulz, Raquel Blazquez, Gero Brockhoff, Tobias Ettl, Mathias Fiedler, Sina Heimer, Juliane Schikora, Richard J. Bauer, Anja Kathrin Wege

**Affiliations:** ^1^ Department of Gynecology and Obstetrics, University Medical Center Regensburg, Regensburg, Germany; ^2^ Bavarian Cancer Research Center (BZKF), Regensburg, Germany; ^3^ Department of Oral and Maxillofacial Surgery, University Hospital Regensburg, Regensburg, Germany; ^4^ Department of Oral and Maxillofacial Surgery, Experimental Oral and Maxillofacial Surgery, Center for Medical Biotechnology, Regensburg, Germany; ^5^ Department of Internal Medicine III, Hematology and Medical Oncology, University Hospital Regensburg, Regensburg, Germany; ^6^ Department of Dermatology, University Hospital Regensburg, Regensburg, Germany; ^7^ Experimental Ophthalmology, University Marburg, Marburg, Germany

**Keywords:** head and neck squamous cell carcinoma (HNSCC), immune checkpoint inhibitors (ICI), tumor microenvironment (TME), multiplexed immune profiling, (patient-derived) organotypic slice co-culture, predictive biomarkers, *ex vivo* culture model, precision oncology

## Abstract

**Background:**

Immune checkpoint inhibitors (ICIs) have become an integral part of cancer therapy, but only a minority of patients experience durable responsiveness. Response rates vary greatly and are often unpredictable, highlighting the urgent need for predictive biomarkers to guide treatment decisions.

**Methods:**

We investigated immune- and tumor-specific expression and secretion profiles in peripheral blood and tumor samples derived from patients with head and neck squamous cell carcinoma (HNSCC). We combined flow cytometry, LEGENDplex™ immune profiling, and preoperative/postoperative serum cytokine analyses to determine checkpoint molecules (e.g., PD-1, TIM-3, LAG-3), immune cell profiles, as well as key markers on tumor cells (CD44, PD-L1, MHC class I/II). In addition, a 3D co-culture model using tumor slices and autologous mononuclear cells from selected HNSCC patients were analyzed upon atezolizumab and pembrolizumab treatment.

**Results:**

Co-expression of PD-1 and TIM-3 on a subset of CD8^+^ tumor-infiltrating T cells was frequently observed, alongside a pronounced infiltration of myeloid cells in the tumor microenvironment. In the peripheral blood, we detected elevated levels of soluble CD27 in patients compared to controls and distinct preoperative cytokine profiles (e.g., reduced IFN-γ, CCL3, CCL20; elevated IL-15/IL-16). Postoperatively, most cytokines showed lower levels compared to healthy controls but significantly higher CCL2 levels. Furthermore, tumor–immune co-cultures from selected patients showed a stronger apoptotic response and phenotypic differences (e.g., increased PD-1 and CD137 expression) upon atezolizumab treatment. Individual changes in soluble factor release (e.g., Gal-9, sPD-L1, sCD25, and sTIM-3) was noticeable upon co-culture under immune checkpoint therapy.

**Conclusions:**

This study provides proof-of-principle data suggesting that a combined multiplexed marker profiling and a functional 3D co-culture assay may help to explore predictive ICI response for HNSCC patients in the future. However, extensive studies with larger cohorts are warranted to validate and refine this approach.

## 1 Introduction

Head and neck squamous cell carcinoma (HNSCC) ranks as one of the most prevalent malignancies globally, presenting a considerable health challenge. Immune checkpoint inhibitors (ICIs) have become a crucial element in the treatment of HNSCC, especially in cases of recurrence or metastasis. Antibodies that target the PD-1/PD-L1 pathway, including pembrolizumab and nivolumab, have received approval for advanced HNSCC and have shown enhanced outcomes in certain patient populations (1). Only a minority of patients, however, show consistent responses to ICIs, which emphasizes the need of consistent predictive biomarkers to spot responders either before or early in therapy (2).

Predicting therapy response in HNSCC presents significant challenges due to tumor heterogeneity and the limitations of existing biomarkers. Intra- and intertumoral heterogeneity can result in considerable variability in the expression of immunological targets such as PD-L1 across different regions of the same tumor or among patients (3).

The predictive value of biomarkers like PD-L1 can be compromised by the heterogeneity of a single tumor biopsy, which can render it unrepresentative of the overall tumor microenvironment (TME). Additionally, variability in detection assays complicates patient stratification. Different immunohistochemical platforms and scoring cut-offs, which range from 1% to 50% PD-L1 positivity, produce inconsistent results, complicating the standardization of PD-L1 as a predictor of ICI benefit (4). Moreover, current diagnostic approaches overlook the complexity of PD-L1 biology, including its isoforms and subcellular localization, despite growing evidence that these factors may influence treatment response and resistance (5).

In addition to PD-L1, various potential markers such as tumor mutational burden, gene expression signatures, and the presence of infiltrating immune cells or soluble cytokines have been investigated (6–8). However, no individual biomarker has demonstrated adequate accuracy for consistent clinical application. The identification of reliable biomarkers that reflect the molecular and immunological diversity of HNSCC is essential for enhancing patient selection and outcomes in ICI therapy.

In the light of these challenges, innovative multi-dimensional strategies are being developed to enhance predictive accuracy for immunotherapy response. Multiplexed immune profiling is a strategy that involves the simultaneous evaluation of multiple immune and tumor parameters from patient samples. It offers a detailed analysis of the TME by simultaneously capturing a wide range of checkpoint molecules, immune cell subset distributions, and cytokine profiles. This comprehensive method may reveal intricate biomarker signatures or combinations that are associated with response, potentially surpassing any individual predictor. Advanced *ex vivo* 3D culture models, in conjunction with molecular profiling, have become significant functional assays for evaluating therapy response in a patient-specific context (9). These models retain the heterogeneity of the original tumor environment including infiltrated immune cells, which enable researchers to examine tumor–immune interactions and the effects of ICIs (10–13) in conditions that facilitate a direct assessment of the functional aspects of response prediction.

Patient-derived tumor slice culture is particularly important among 3D culture methods because it can maintain the natural architecture and cellular heterogeneity of the tumor. Unlike conventional 2D cell lines or dissociated organoids, thin sliced tumor explants preserve the original tumor–stromal context including cancer cells, supporting stromal cells, and resident immune infiltrates (11). Crucially, immune cells in these slices remain alive and functional, hence immunotherapeutic agents can cause responses (e.g., T cell activation or tumor cell killing) measurable *ex vivo* (14). 3D tumor slice models offer a modern platform to functionally test ICIs and track biomarkers of response or resistance in real time and short time by recapitulating important elements of tumor heterogeneity and immune engagement. Autologous peripheral blood mononucleated cells (PBMCs) can be added to this organotypic system to replicate immune cell penetration, so producing a miniaturized TME in the laboratory. However, this has been only occasionally investigated, e.g., using autologous spleenocytes or PBMCs proving immune cell infiltration into the tissue slices (15).

In this study, we established an *ex vivo* tumor response explorative model that combines a 3D tumor slice co-culture assay with multiplexed immune profiling to enhance the prediction of ICI therapy response in HNSCC. We integrate comprehensive immune and tumor phenotyping, which encompasses the expression of various checkpoint receptors on tumor-infiltrating lymphocytes, the status of immunoregulatory ligands on tumor cells, and systemic cytokine levels, with a patient-specific functional assay utilizing HNSCC tumor slices co-cultured with autologous immune cells. This integrated approach enables the assessment of both baseline immunological characteristics of the tumor and the dynamic response of the tumor–immune cell ensemble to checkpoint blockade. In our co-culture experiments, tumor slices are subjected to PD-1 blockade (pembrolizumab) and PD-L1 blockade (atezolizumab), facilitating a direct comparison of their functional effects on the TME. We aim to identify biomarker signatures that differentiate ICI-responsive tumors from non-responsive ones by correlating these *ex vivo* responses with the multiplexed profiles. This proof-of-concept study indicates that a combined multiplexed marker analysis and 3D co-culture model can function as a more valuable exploratory tool for immunotherapy outcomes in HNSCC, thereby facilitating the development of enhanced personalized and effective treatment strategies.

## 2 Methods

### 2.1 Human tumor and blood sample preparation

HNSCC tumors (detailed summarized in **Table 1**, study 1) were cut into small pieces and digested with collagenase and DNase I (Sigma Aldrich, St. Louis, MO, USA) at 37°C for 30 to 45 minutes (min). Subsequently, tissues were passed through a pre-wetted 40 µm cell strainer (Falcon, Thermo Fisher Scientific, USA) to obtain single cell suspension. Upon centrifugation (300 x g for 5 min at 4°C) supernatant was discarded and cells eluated in 1% fetal calf serum (FCS), 0.01% NaN_3_ and Dulbecco´s phosphate buffered saline (DPBS) buffer (Gibco, Thermo Fisher Scientific, USA). 100 µl of peripheral EDTA blood samples were lysed using FACS lysing solution (BD Biosciences, USA, Cat. No. 349202) and washed twice with 1% FCS, 0.01% NaN_3_ and DPBS buffer (300 x g for 5 min at 4°C).

**Table 1:** Head and neck squamous cell cancer patient specific characteristics.

Variable	HNSCC (study 1)	HNSCC (study 2)
	(n = 10) #	(n = 19) #
Mean age – (yr)	62.5	67.2
Sex - no. (n/%)		
Male	8 (80)	12 (63)
Female	2 (20)	7 (37)
N stage (n/%)		
Nx	1 (10)	3 (16)
N0	4 (40)	9 (47)
N1	1 (10)	1 (5)
N2	2 (20)	3 (16)
N3	2 (20)	3 (16)
M0/M1 (n/%)		
M0	9 (90)	19 (100)
M1	1 (10)	0 (0)
T stage (n/%)		
T1	0 (0)	5 (26.3)
T2	2 (20)	9 (47.4)
T3	4 (40)	2 (10.5)
T4	4 (40)	3 (15.8)
Grade (n/%)		
G1	2 (20)	1 (5)
G2	6 (60)	11 (61)
G3	2 (20)	6 (32)
Clinico-patholocical markers		
p16, HPV^+^ (n/%)	1 (10)	1 (5)
Treatment (n/%)		
Surgery	4 (40)	11 (57.9)
Surgery + RT	3 (30)	5 (26.3)
Surgery + RCT	3 (30)	3 (15.8)

Tumor staging was performed according to the TNM classification system, 8th edition of the UICC (2017), using pathological staging (pTNM). HPV, human papilloma virus; RT, radiation; RCT, radiation + chemotherapy treatment.

In addition, pre- and postoperative serum samples were collected from 19 HNSCC patients (detailed summarized in **Table 1**, study 2) and 20 age-/sex-matched healthy donors. Cytokine levels were quantified via multiplex cytokine analysis. Serum concentrations of IFN-γ, CCL2, CCL3, CCL20, IL-16, SCF, IL-15, CXCL1, LIF, TNF-β, TWEAK, VEGFA, and APRIL were measured using the ProcartaPlex™ Human Immune Monitoring Panel 65plex (Thermo Fisher Scientific, USA) with a Bio-Plex 200 system, following the manufacturer’s protocol. Data were normalized to healthy controls and analyzed via two-way ANOVA with Tukey’s *post-hoc* test.

### 2.2 Flow cytometry

Tumor single cells and peripheral blood samples of the respective patients were stained with the following fluorochrome labeled antibodies purchased from BioLegend (San Diego, CA, USA): αCD45-BV510 (304036, HI30, RRID: AB_2561940), αCD8a-BV510 (301048, RPA-T8, RRID: AB_2561942), αCD33-PerCP-Cy5.5 (303414, WM53, RRID: AB_2074241), αCD19-PE (363004, SJ25C1, RRID: AB_2564126), αPD-1-AF647 (329910, EH12.2H7, RRID: AB_940471), αPD-L1-BV421 (329714, 29E2A3, RRID: AB_2563852), αEpCAM-AF647 (324212, 9C4, RRID: AB_756086), αEGFR-AF488 (352908, AY13, RRID: AB_11126165), αCD56-PeCy7 (362510, 51H11, RRID: AB_2563927), αCD44 (103044, IM7, RRID: AB_2650923), αCD45-PerCP-Cy5.5 (304028, HI30, RRID: AB_893338), αCD137-PeCy7 (309818, 4B4-1, RRID: AB_2207741), αTIM-3-BV421 (345008, F38-2E2, RRID: AB_11218598). From BD Biosciences (San Jose, CA, USA), we purchased αCD3-FITC (555332, UCHT1, RRID: AB_395739), and αCD4-APC-H7 (641398, SK3, RRID: AB_1645732), αMHCII-BB700 (742224, Tu39, RRID: AB_2871434). The following antibodies were used for staining from eBiosciences, Thermo Fisher Scientific: αCD24-PECy7 (25-0247-42, eBioSN3 SN3 A5-2H10, RRID: AB_2573334), αMHCI-PE (MA1-10346, MEM-123, RRID: AB_11154825). The antibody αLAG-3-PE (FAB2319P, polyclonal goat IgG, RRID: AB_2133351) was ordered from R&D Systems (Minneapolis, MN, USA). Gating strategy is displayed in **Supplementary Figure 1**. For all flow cytometry panels, gates were set using appropriate isotype controls to define marker expression. Software Single cells from tumor and blood were incubated for 30 min at 4°C and subsequently washed twice (300 x g, 5 min, 4°C) with DPBS containing 1% FCS and 0.01% NaN_3_. Protein expression profiles of tumor and immune cells were analyzed by flow cytometry with a FACS-Canto-II (BD Biosciences, San Jose, CA, USA), which was run by the BD FACSDiva™ software 7.0 (BD Biosciences, San Jose, CA, USA). Results were analyzed using the FlowJo™ software v10.8 (BD Biosciences, San Jose, CA, USA).

### 2.3 Soluble factors analyzed by LEGENDplex™ bead-based immunoassay

EDTA blood samples were centrifuged (2000 x g, 10 min, at 4°C) and supernatant plasma stored at -80°C. Multiplex assay procedure of LEGENDplex™ 12-plex HU Immune Checkpoint Panel 1 (Cat No. 740867, analyzed molecules: sCD25, 4-1BB, sCD27, B7.2, free active TGF-ß1, sCTLA-4, sPD-L1, sPD-L2, sPD-1, sTIM-3, sLAG-3, and sGalectin-9) was performed as described in the manufacturer´s protocol. Briefly, human plasma samples were pre-diluted and were incubated with microbeads (800 rpm; 2 hours, room temperature), washed and incubated with detection antibody (800 rpm, 1 hour, room temperature) followed by Streptavidin-PE (SA-PE) incubation (800 rpm, 30 min, room temperature). Data were analyzed with the LEGENDplex™ Data Analysis Software Suite (BioLegend, San Diego, CA, USA).

### 2.4 3D co-cultivation of HNSCC tumor slices with autologous peripheral blood derived mononuclear cells (MNCs)

HNSCC tumors were immediately stored in DMEM medium after surgery (Gibco, Thermo Fisher Scientific, USA) containing 10% fetal calf serum (Gibco, Thermo Fisher Scientific, USA) and 1% penicillin-streptomycin (10.000 U/mL; 10 mg/mL) (P/S); Sigma-Aldrich) and 1% amphotericin (250µg/mL; Sigma-Aldrich) on ice. Subsequently, tumors from six individual patients from study group 1 were cut into 350 µm slices using a vibratome (Leica VT1200S) as described in detail before (16). Autologous MNCs were isolated from peripheral EDTA patient blood. Samples were pre-diluted 1:1 with DPBS, transferred on top of a 20 mL Pancoll (PAN-Biotech, Germany) solution and centrifuged (600 x g; 20 min, room temperature; w/o brake). Samples were centrifuged (600 x g; 20 min, room temperature; w/o brake). Cells from buffy coat were transferred into DPBS solution, counted and centrifuged (300 x g; 5 min, room temperature). Subsequently, supernatant was discarded and MNCs were resuspended in pre-warmed culture medium (see above). 0.5 x 10^6^ MNCs were added to each well containing tumor tissue slice. For treatment, 5 µg/mL atezolizumab or 10 µg/mL pembrolizumab were supplemented. For each patient and condition, multiple tumor slices (typically 3-5, as available based on tissue size) were pooled to compensate inter-slice variations. After six to seven days slices were digested for 30–45 min using 1 mg/mL collagenase and 20 µg/mL DNase I at 37°C. Tissue was passed through a 40 µm cell strainer and washed twice. Multicolor staining was performed as described above. In addition, apoptosis induction was determined by FITC-Annexin V (Immunotools) and DAPI (Sigma-Aldrich) staining.

### 2.5 Statistical analyses

All results are shown as mean or median and standard deviation (SD), as described in the figure legends. Statistical analyses were performed using GraphPad PRISM 8. Data were determined to be statistically significant if p ≤ 0.05 according to multiple T-test, or if data were non-parametric Mann-Whitney U test or Kruskal-Wallis test with Dunn´s multiple comparisons test. Correlations were determined using two-tailed Pearson correlation test. Detailed information is included in the figure legend and asterisks denote statistical significance (* p ≤ 0.05, ** p ≤ 0.01, *** p ≤ 0.001; **** p ≤ 0.0001).

### 2.6 Ethics statement

All patients have signed a written informed consent. Patient-derived tumor samples and peripheral blood samples were taken with approval from ethics committee of University of Regensburg (# 23-3161-101). Informed consent was obtained from all individual participants included in the study.

## 3 Results

### 3.1 Individual differences in tumor and immune cell phenotype of HNSCC patients

**Figure 1:**
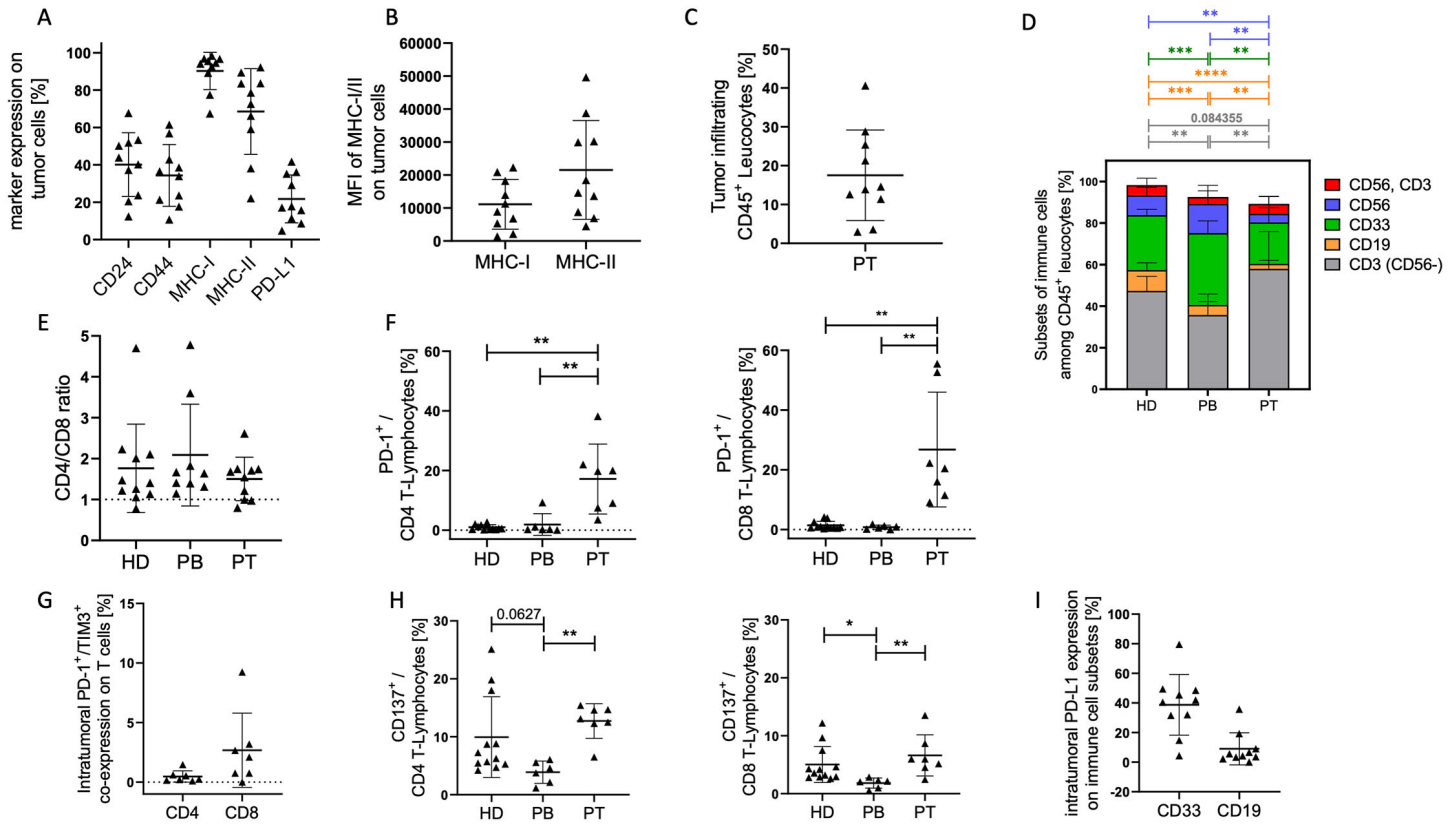
Tumor cell characterization in HNSCC samples. EGFR/EpCAM^+^ tumor cells were analyzed by flow cytometry. **(A)** Graphs represent the proportion of stem cell associated markers CD24^+^ and CD44^+^, as well as antigen-presenting molecules MHC-I^+^ and MHC-II^+^, and the PD-L1^+^ checkpoint molecule on tumor cells from HNSCC patients (**Table 1**; study 1; n = 10). The mean fluorescence intensities (MFI) of MHC-I and II **(B)** and the overall immune cell infiltration **(C)** are displayed. Each symbol represents a single donor; **(D)** The graph summarizes the mean proportions of T lymphocytes (grey), B lymphocytes (orange), myeloid cells (green), NK cells (blue), NK-T cells (red) in tumor samples (PT), blood of patientss (PB), and healthy controls (HD). The ratio of CD4/CD8 **(E)**, the PD-1 **(F)**, PD-1*/*TIM-3 co-expression **(G)**, and CD137 **(H)** expression on CD4^+^ and CD8^+^ T cells are displayed. **(I)** Graph represents the expression of PD-L1 on myeloid (CD33^+^) and B (CD19^+^) cells. Each symbol represents one individual patient. Data are shown as mean +/-SD and p-values were calculated by Kruskal-Wallis test (Dunn´s multiple comparisons test) or multiple t-test based on parametric pretesting; * p ≤ 0.05; ** p ≤ 0.01; *** ≤ p 0,001; **** ≤ p 0,0001.

Individual differences of the stem cell associated markers CD24 and CD44, as well as MHC- and PD-L1 expression on tumor cells of HNSCC patients were detectable using flow cytometry (**Figure 1A**). The data suggest that the analyzed HNSCC tumors are richly populated by tumor cells expressing both MHC-I (~95%) and MHC-II (~70%) as well as PD-L1 (~20%). Among MHC-I and MHC-II there was a wide range of expression intensity (MFI; **Figure 1B**). In addition, individual differences in the proportion of infiltrating immune cells were found (**Figure 1C**). The TME contained a notable fraction of infiltrating leukocytes (mean of CD45^+^ ~18%). Overall, the peripheral blood from HNSCC patients (PB) contained an increased myeloid and a decreased B and T cell proportion (**Figure 1D**) compared to healthy donors (HD) as controls. Furthermore, tumor infiltrating immune cells in the patient tumor tissue (PT) contained an increased T cell and decreased proportion of myeloid, B, and NK (CD56^+^) cell proportion compared to these subsets in the patients’ blood (PB). In summary, the majority of immune cells in the tumor belonged to the T cell subsets but complemented with 20% (mean ± SD 12.4) myeloid cells and a small proportion of B and NK/NK-T cells. There was no difference in CD4/CD8 ratio between healthy donor or HNSCC PB or their tumors (**Figure 1E**). However, T cells exhibited a significant higher expression of PD-1 (mean on CD4^+^: 17% ± 12 SD; mean on CD8^+^: 27% ± 19 SD) on tumor infiltrating lymphocytes (TIL) compared to healthy donors or patient blood (**Figure 1F**; CD4^+^ = p = 0.004; CD8^+^ = p = 0.002). A subset of these PD-1^+^ T cells also co-expressed TIM-3 (mean on CD4^+^: 0.48% ± 0.5 SD; mean on CD8^+^: 2.67% ± 3.11 SD), suggesting an “exhausted” phenotype (**Figure 1G**). CD137 expression was significantly lower in CD4^+^ (p = 0.032) and CD8^+^ (p = 0.019) T cells in the PB compared to TIL and also reduced compared to healthy control (**Figure 1H**; CD4^+^ = p = 0.063; CD8^+^ = p = 0.012). About 40% of myeloid cells (CD33^+^) and ~10% of B cells (CD19^+^) also expressed PD-L1 (**Figure 1I**), reinforcing a multifaceted immunosuppressive network within the tumor.

Overall, the data paint a picture of an immune-active yet checkpoint-inhibited TME, where both tumor cells and immune cells contribute to immunosuppression through PD-L1 expression and T cell PD-1/TIM-3 (co-)expression. These findings underscore the rationale for immune combination checkpoint blockade strategies in HNSCC.

### 3.2 Individual differences in the secretion of soluble factors in the plasma of HNSCC patients

**Figure 2:**
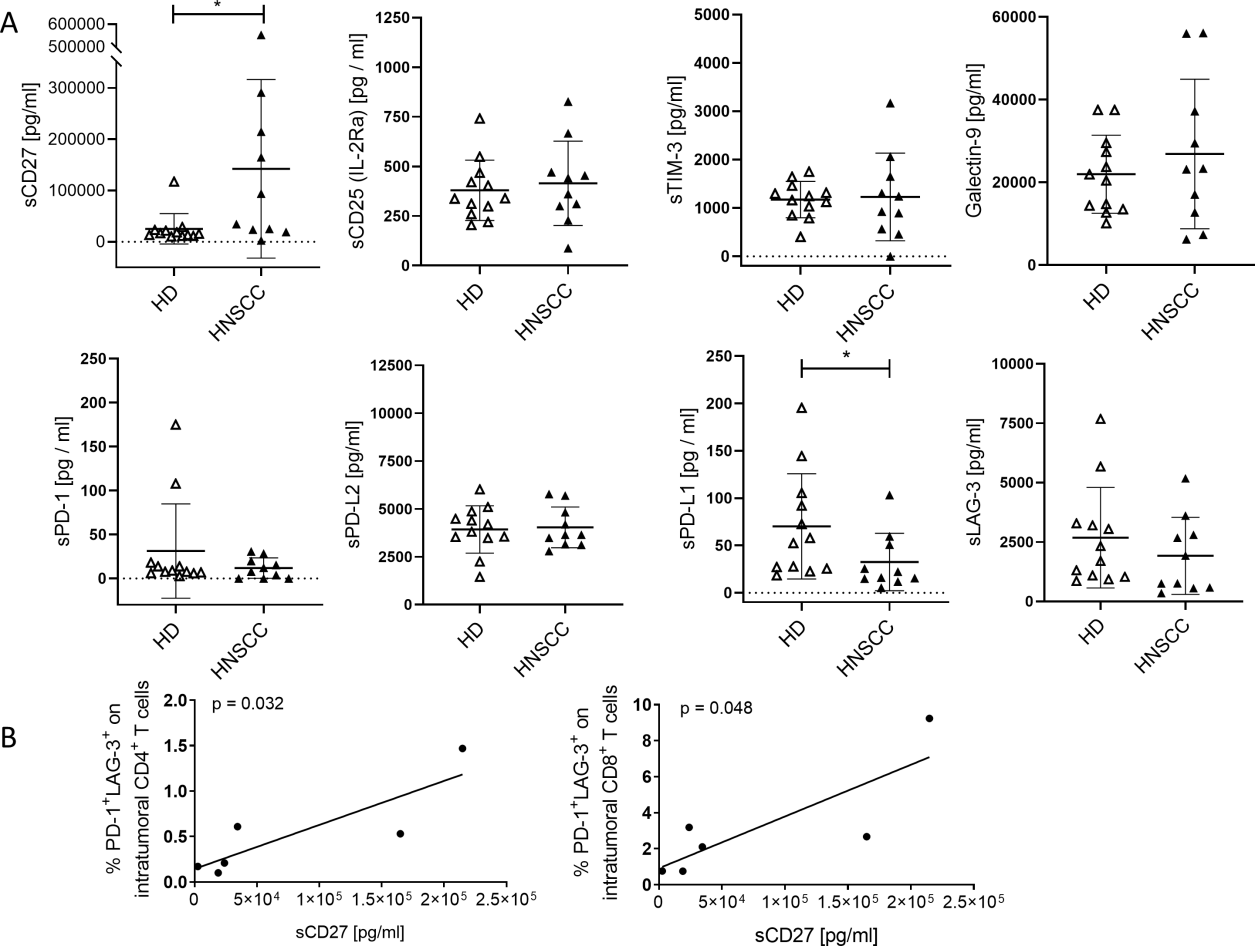
Soluble immune checkpoints molecules and related regulatory factors in plasma of HNSCC patients compared to healthy controls anlyzed by LEGENDplex*™*. **(A)** Concentrations of soluble immune activation markers (sCD27, sCD25), soluble immune checkpoint molecules (sTIM-3, sPD-1, sLAG-3, sPD-L1, sPD-L2), and the immunoregulatory factor galectin-9 were analyzed in plasma of cancer patients (HNSCC, **Table 1**; study 1; n = 10) and healthy donors (HD). Each symbol represents one individual patient. Data are given as mean ± SD and analyzed by unpaired Student*’*s t-test**;** *p ≤ 0.05. **(B)** Correlations of sCD27 to proportion of PD-1^+^LAG-3^+^ CD4 and CD8 T cells were determined. Correlation were determined using the two-tailed Pearson correlation test and p-values are indicated in each graph.

**Figure 2** shows a statistically significant increase in the mean sCD27 concentration in plasma samples from HNSCC patients (mean: 142214 pg/mL ± 174038 SD) compared to healthy donors (HD; mean: 25139 pg/mL ± 29583 SD). In contrast, sPD-L1 was significantly reduced in HNSCC (mean: 32.53 pg/mL ± 30.3 SD) compared to healthy controls (mean: 70.16 pg/mL ± 55.6 SD). Other soluble checkpoint molecules and regulatory factors, including sCD25, sTIM-3, Galectin-9, sPD-1, sPD-L2, and sLAG-3, revealed no significant differences between the two groups. These findings suggest a notable increase in mean sCD27—potentially reflecting enhanced T cell activation or turnover—alongside with a reduction in sPD-L1, which may indicate altered regulatory pathways or a shift toward cell-bound PD-L1 expression in HNSCC. Furthermore, there is a correlation between sCD27 and the proportion of PD-1^+^LAG-3^+^ CD4^+^ (p = 0.032; r = 0.85) and CD8^+^ T cells (p = 0.048; r = 0.82) detectable (**Figure 2B**).

**Figure 3:**
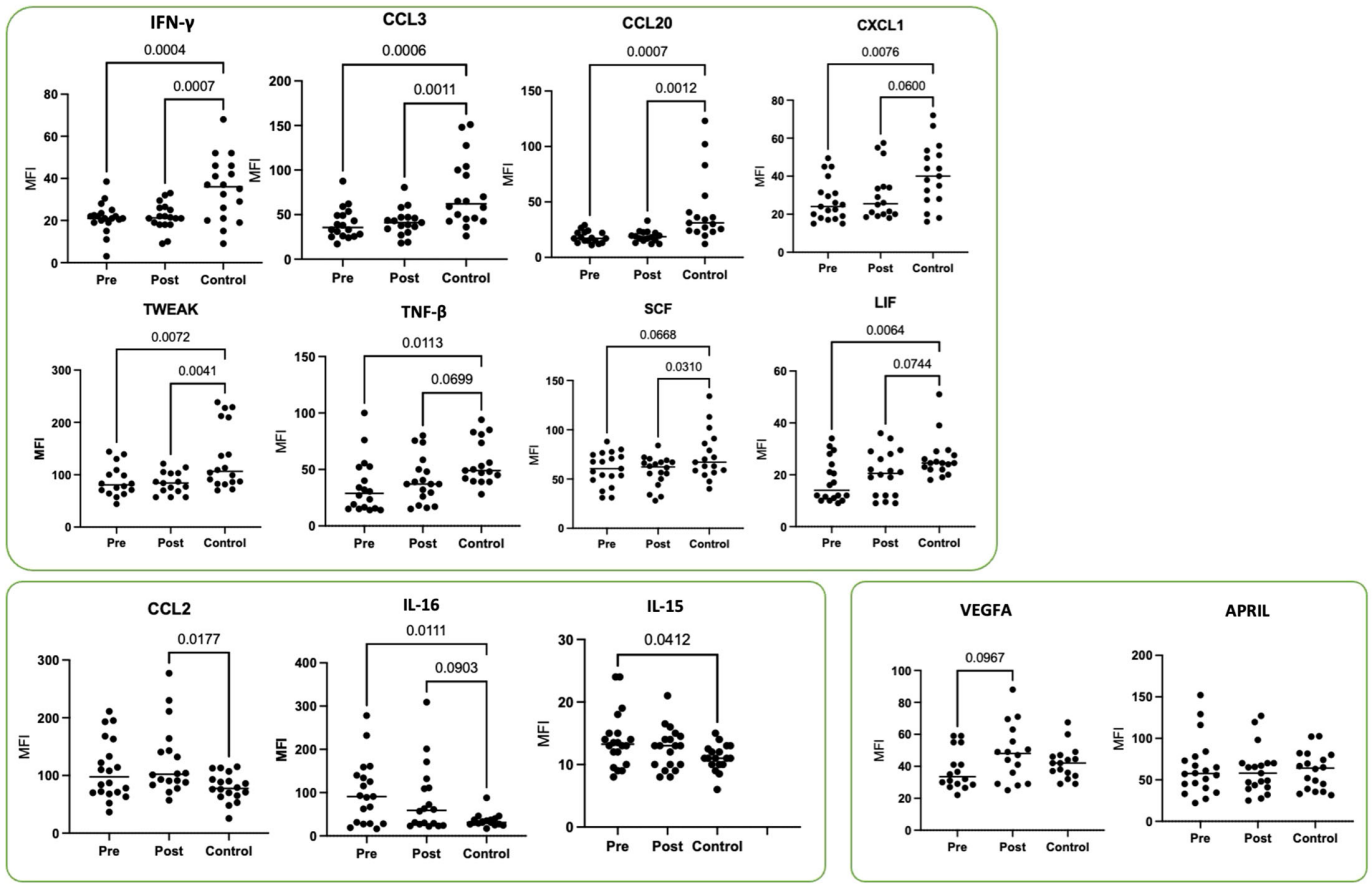
Plasma cytokine profiles in HNSCC patients before (pre) and 4–6 weeks after (post) surgical resection compared to age-matched healthy controls. Shown are the mean fluorescence intensity (MFI) values for IFN-γ, CCL3, CCL20, CXCL1, TWEAK, TNF-β, SCF, LIF, CCL2, IL-16, IL-15, VEGFA, and APRIL from HNSCC patients (**Table 1**; study 2; n = 19) and healthy donors as controls (Control, n = 20). Each symbol represents one individual patient. Data were normalized to healthy controls and analyzed via two-way ANOVA with Tukey’s *post-hoc* test.

Moreover, we assessed the plasma levels of multiple cytokines and chemokines in HNSCC patients before (pre) and 4–6 weeks after (post) surgical resection and compared them to an age-matched healthy control group (Control) (**Figure 3**). Overall, most cytokines were either suppressed (e.g., IFN-γ, CCL3, CCL20, CXCL1, TWEAK, TGF-β, SCF, LIF) in PB from HNSCC patients compared to controls or showed an elevated trend (e.g., CCL2, IL-16, IL-15) at specific time points. Notably, the levels of VEGFA and APRIL remained unchanged among all groups.

### 3.3 Increased apoptosis upon atezolizumab treatment

**Figure 4:**
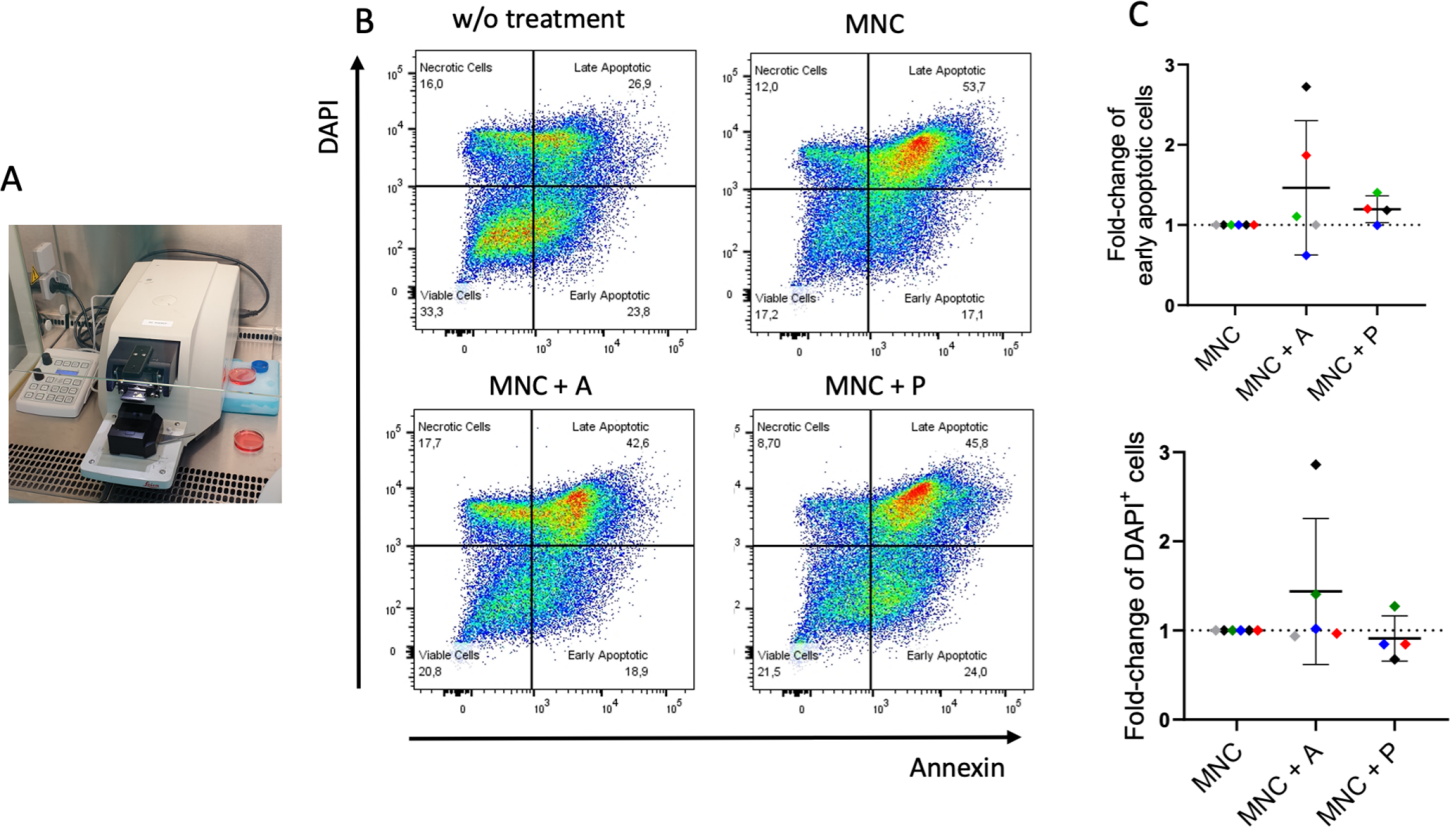
Ex-vivo 3D co-culture of HNSCC tumor slices with autologous mononuclear cells (MNC) ± immune checkpoint treatment. **(A)** Tissue slices from six HNSCC (**Table 1**; study 1) were generated by a vibratome and co-cultured with autologous MNCs from the patients for six or seven days. **(B)** Gating strategy for the detection of apoptosis using Annexin V and DAPI. Plots represent tumor slices without MNC and treatment (w/o treatment), tumor slices with autologous MNCs (MNC; n = 6), and with atezolizumab (MNC + A; n = 5) or pembrolizumab (MNC + P; n = 4) treatment. **(C)** Fold-change of early apoptotic (Annexin V^+^, DAPI^–^) and late*/*very late apoptotic (DAPI^+^) cells of tumor slices incubated with MNC with atezolizumab (MNC + A) or pembrolizumab (MNC + P) in comparison to MNC. Each color represents tumor samples derived from the same patient. Data are given as mean ± SD.

*Ex-vivo* 3D tissue slices from HNSCC tumors were co-cultured with autologous MNCs and treated with atezolizumab or pembrolizumab (**Figure 4**). Using Annexin V and DAPI staining to assess apoptosis via flow cytometry, we observed increased early and late apoptotic cell fractions in selected donors upon atezolizumab (anti-PD-L1) but not pembrolizumab (anti-PD-1) treatment (**Figures 4B, C**). Of note, the strongest response to atezulizumab was detected in a patient, who already died without receiving ICI (black symbol; **Figure 4C**).

### 3.4 Individual changes in expression and secretion profiles of 3D co-cultured HNSCC samples

**Figure 5:**
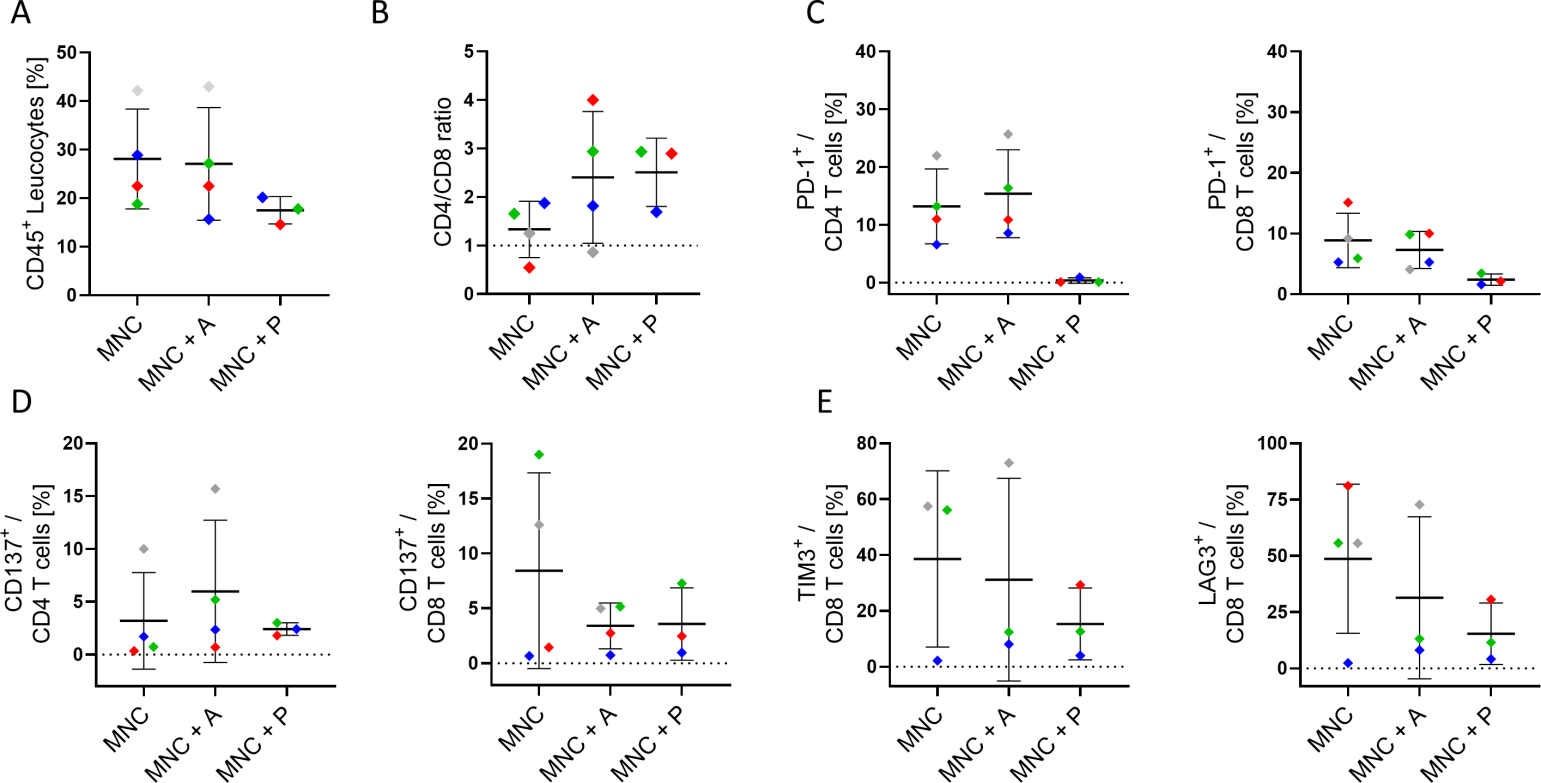
Characterization of infiltrating immune cells in 3D co-culture of HNSCC tumor slices. **(A)** Overall CD45^+^ leukocyte infiltration, **(B)** CD4/CD8 ratio, **(C)** PD-1 expression (on CD4^+^ & CD8^+^), **(D)** CD137 expression (on CD4^+^ & CD8^+^), and **(E)** TIM-3 and LAG-3 expression (on CD8^+^ T cells) in the tumor tissue of four HNSCC patients (**Table 1**; study 1) after six to seven days of culture with autologous mononuclear cells (MNC), or with MNCs and atezolizumab/pembrolizumab treatment (MNC + A, MNC + P) were analyzed. Each color represents tumor samples derived from the same patient. Data are given as mean ± SD.

**Figure 6:**
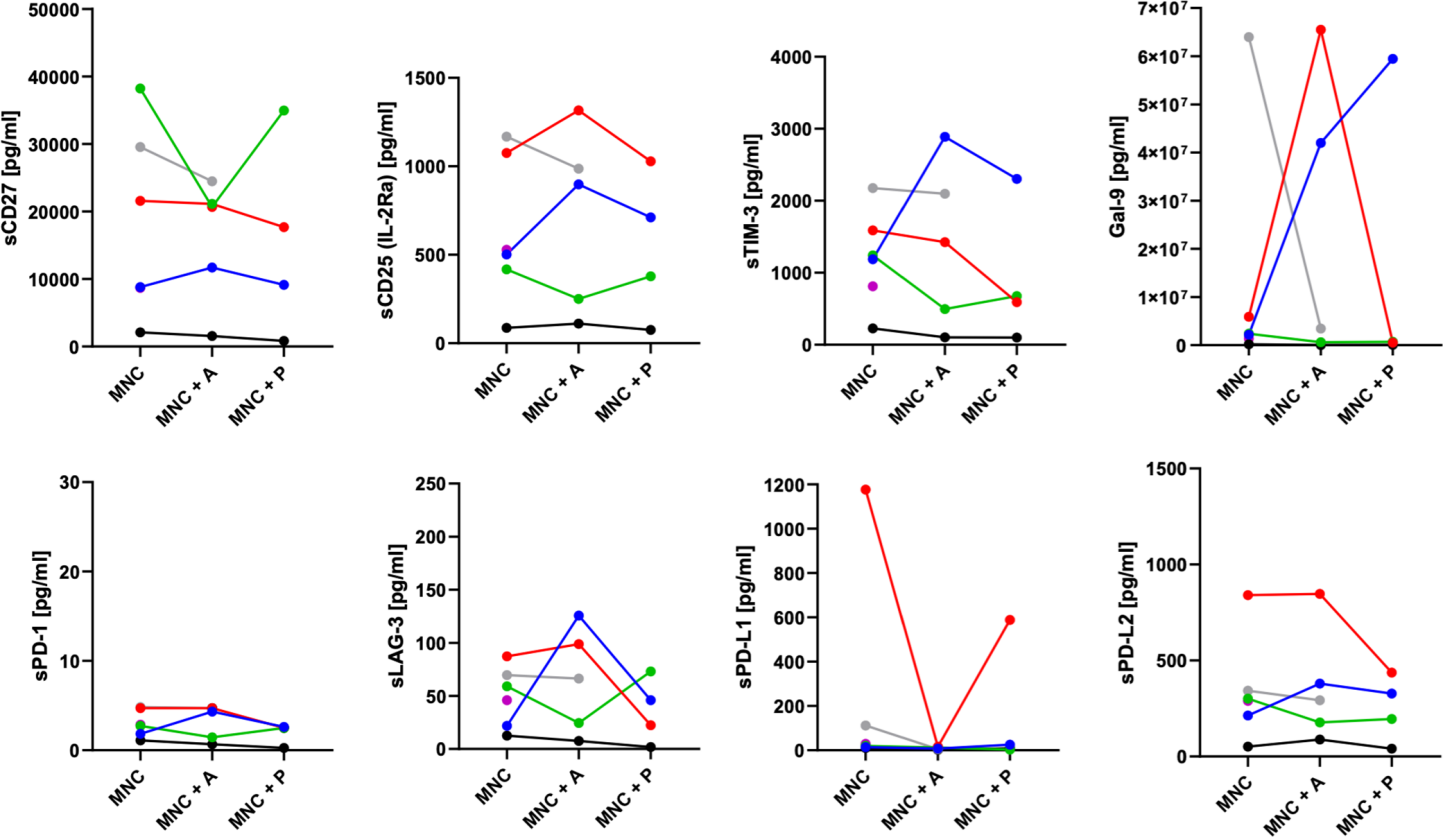
Release of soluble checkpoints and regulatory factors in the supernatant of HNSCC 3D co-cultures. Concentrations of soluble immune activation markers (sCD27, sCD25), soluble immune checkpoint molecules (sTIM-3, sPD-1, sLAG-3, sPD-L1, sPD-L2), and the immunoregulatory factor galectin-9 from up to six HNSCC tumor patients (**Table 1**; study 1) were analyzed by LEGENDplex™ upon six to seven days of co-cultivation with autologous mononuclear cells (MNC), or with MNCs and atezolizumab/pembrolizumab treatment (MNC + A, MNC + P). Each color represents tumor samples derived from the same patient.

In parallel to the apoptosis assessment, flow cytometric characterization of infiltrating immune cells was performed on the *ex-vivo* 3D tissue slice cultures upon treatment (**Figure 5**). Flow cytometric characterization of infiltrating immune cells (**Figure 5**) revealed individual variations in the overall CD45^+^ leukocyte infiltration (**Figure 5A**), the CD4/CD8 ratio (**Figure 5B**), the PD-1 (**Figure 5C**), and the co-stimulatory marker CD137 (**Figure 5D**) expression on CD4^+^ and CD8^+^ T cells depending on the treatment condition. Of note, the reduced detection of PD-1 expression in pembrolizumab treated samples (**Figure 5C**) is potentially due to the masking of the molecule by the anti-PD-1 binding ICI, which hinders anti-PD-1 staining (data not shown). Supernatants from these cultures were collected and revealed an individual secretion profile of specific soluble factors (**Figure 6**). The concentration of sCD27, sCD25, sTIM-3, Gal-9, sPD-L1 and PD-L2 varied considerably between the donors. Moreover, the concentration of nearly all tested molecules increased or decreased individually upon different ICI treatments. The overall concentration of sPD-1 was low in all samples independent of treatment and comparable to HNSCC patient plasma (**Figure 6**). Of note, the reduced detection of sPD-L1 in the atezolizumab treated sample, which exhibit high levels of sPD-L1 if incubated without ICI or pembrolizumab (red symbol) is possibly due to the masking of the molecule by the anti-PD-L1 binding ICI, which hinders anti-PD-1 staining (data not shown).

## 4 Discussion

Our analysis indicates that primary HNSCC tumors are both immunologically active, evidenced by immune cell infiltration, increased PD-1 expression on CD4^+^ & CD8^+^ infiltrating T cells, and the killing capacity *ex vivo*-and immunosuppressed, as shown by substantial PD-L1 positivity (~20% of tumor cells) and a considerable infiltration of myeloid cells with high PD-L1 expression. These findings together with high MHC-I (~95%) and notable MHC-II (~70%) expression align with published literature showing that HNSCC can retain robust antigen-presentation capacity while simultaneously upregulating inhibitory ligands such as PD-L1 to evade cytotoxic T cell responses (17).

Individual differences in the expression profile of CD24 and CD44 were noticeable. This is of importance because CD24 and CD44 have been implicated in HNSCC biology, with CD44 often considered a canonical cancer stem cell (CSC) marker, and the CD24^+^/CD44^+^ phenotype frequently investigated for its association with stemness, aggressive tumor behavior, and therapy resistance in HNSCC and other malignancies (18). Therefore, both markers might serve as a potent biomarker to characterize malignancy in individual patients. Furthermore, the dual expression of CD44 and CD24 is crucial for understanding HNSCC biology and developing targeted therapies. Given CD44’s role in chemotherapy resistance, strategies aiming to disrupt CD44 signaling could enhance treatment efficacy (19, 20).

The detection of a significant CD45^+^ population (~18% of all cells) dominated by T cells mirrors previous reports of T-cell-rich HNSCC microenvironments (21, 22). However, our observation that ~20% of CD4^+^ and ~19% of CD8^+^ T cells express PD-1, with a fraction co-expressing TIM-3, suggests a phenotype, which is also considered as T-cell exhaustion. These data reinforce prior findings that co-expression of multiple checkpoints (PD-1, TIM-3, LAG-3) frequently marks dysfunctional TILs (23). Notably, TIM-3 upregulation can emerge as an adaptive resistance mechanism when PD-1 is therapeutically blocked, underscoring the potential need for dual checkpoint inhibition (24).

The higher levels of CD137 (4-1BB) on T cells in the tumor relative to peripheral blood highlight a paradox: T cells display signs of both activation and inhibition within HNSCC (25). Recent work has shown that “partially exhausted” T cells can still be reactivated under certain conditions—particularly if dominant inhibitory receptors like PD-1 and TIM-3 are therapeutically targeted. Thus, our finding of CD137^+^ T cells suggests that there is a residual capacity for tumor-specific T-cell responses that combination immunotherapy could harness.

Our data demonstrate that a substantial proportion of myeloid (CD33^+^) cells (~40%) and a smaller fraction of B cells (~10%) also express PD-L1. This is consistent with studies indicating that not only tumor cells but also multiple immune subsets help maintain an immunosuppressive microenvironment in HNSCC (21, 26). Myeloid-derived suppressor cells and other myeloid cells can produce immunosuppressive factors and upregulate PD-L1, further dampening T cell function. The impact of myeloid cells in HNSCC has been previously described. Among various myeloid subsets, M2 macrophages, the main population of tumor-associated macrophages (TAMs), exhibit immune suppressive functions characterized, for example, by their local production of IL-10 or TGF-β (27). In HNSCC patients M2 TAM and higher levels of TGF-β were identified (28), and high levels of TAMs have been correlated to tumor progression and metastases formation in HNSCC (29, 30). In a squamous cancer mouse model, the depletion of macrophages inhibited tumor growth and TAM infiltration (31). Furthermore, TAMs seems to be involved in ICI resistance (32) and therefore TAM inhibition by various strategies (33) in combination with checkpoint therapies are ongoing in different clinical trials (34). For instance, in a 3D co-culture of autologous immune cells with PD-L1^+^ gastric cancer organoids, unresponsiveness to anti-PD-1 therapy was observed in the presence of myeloid-derived suppressor cells (MDSCs); conversely, MDSC depletion enhanced anti-PD-1 mediated organoid killing (35).

Our data indicate a distinctive shift in soluble immune checkpoint molecules in HNSCC, most notably the significant increase in sCD27 and a decrease in sPD-L1, with other mediators (sCD25, sTIM-3, Galectin-9, sPD-1, sPD-L2, sLAG-3) remaining relatively unchanged. These findings resonate with recent investigations showing that while soluble checkpoint molecules can reflect overall immune activation or suppression, not all checkpoints exhibit uniform alteration in HNSCC (6, 23). Although group-level differences in several biomarkers were statistically significant, considerable inter-patient heterogeneity and overlapping values between groups were observed. This emphasizes the complex nature of the tumor immune microenvironment and the challenge in finding single biomarkers that have high predictive value for each individual.

CD27 is a co-stimulatory immune checkpoint found on various immune cells, predominantly T cells. When it binds to its ligand CD70, which is primarily expressed by antigen-presenting cells, it triggers a signaling cascade that promotes T cell activation and proliferation (36). Upon activation, matrix metalloproteinases cleave CD27, resulting in the release of soluble (s)CD27. Unlike its membrane-bound counterpart, which serves as a co-stimulatory molecule, sCD27 appears to elicit inhibitory activity associated with tumor progression and immunosuppression (37). In context of immunotherapy, lower levels of sCD27 have been associated with longer progression-free survival (PFS) or overall survival (OS) in different solid cancers (38).

In the samples analyzed here, a correlation of increased sCD27 with enhanced proportion of PD-1^+^LAG-3^+^ CD4^+^ and CD8^+^ T cells were found, suggesting a more exhausted T cell state (39).

PD-L1 is present not only on tumor cells but also on various other cell types, including immune cells such, e.g., B cells, myeloid cells (**Figure 1**), and T cells. However, its secretion appears to be restricted to myeloid cells (40) suggesting a different mechanism in the regulation process of expression and shedding. Soluble PD-L1 has been described to induce T cell apoptosis and thereby can compete with the inhibitory effect of mPD-L1 (41). Moreover, sPD-L1 also affects macrophages guiding their polarization toward an inhibitory function (42). Mostly, increased levels of sPD-L1 have been associated with disease progression, poorer outcomes across various cancer subtypes and failure upon checkpoint therapy (43). A reduced sPD-L1 in the circulation is compatible with reports suggesting that HNSCC often localizes PD-L1 expression to the TME, thereby concentrating immunosuppressive signaling to the tumor-immune interface rather than to a systemic phenomenon (6, 44). These studies have further shown that high membrane-bound or exosomal PD-L1 is a primary driver of T cell dysfunction in HNSCC, while sPD-L1 levels may not necessarily reflect disease progression or recurrence (45). Consequently, the observed decrease in sPD-L1 could underscore a shift toward cell-surface PD-L1 retention or a diminished need for shedding, which in turn might limit systemic immune modulation. However, the exact mechanisms and clinical implications of sPD-L1 in cancer progression and during ICI therapy remain an active area of research.

Notably, soluble levels of other checkpoints, including sTIM-3, sPD-1, and sLAG-3, remained unchanged, suggesting that local but not systemic checkpoints may shape immune dysregulation in HNSCC. Prior research similarly indicates that while TIM-3 and LAG-3 co-expression on T cells strongly influences local T cell exhaustion, their soluble forms do not consistently correlate with clinical outcomes in HNSCC (24).

Furthermore, even if soluble markers like sCD27 and sPD-L1 show statistically significant changes, several obstacles have to be solved before they can be directly applied into the clinic. Among these are defining clinically significant cut-off values, establishing standardized assay protocols and platforms to guarantee inter-laboratory repeatability, and mandating validation in bigger, prospective, and uniformly treated patient cohorts. Beyond tumor-specific immunity, various physiological and pathological conditions can also affect the dynamic nature of these soluble factors. Therefore, it is crucial to interpret findings carefully, considering each patient’s unique characteristics and the disease’s progression stage.

Our study highlights a persistently dysregulated cytokine milieu in HNSCC patients, marked by decreased pro-inflammatory and immunomodulatory mediators (IFN-γ, TNF-β, CCL3, CCL20, CXCL1, TWEAK, SCF, and LIF) compared to healthy controls. These findings are in line with broader investigations documenting tumor-mediated immunosuppression in head and neck cancers (46). Notably, this reduced cytokine landscape was evident both before and 4–6 weeks after surgical resection, supporting the notion that HNSCC-associated immune suppression does not fully normalize in the early postoperative period. The reduced levels of key cytokines, such as IFN-γ, have been linked to diminished T cell effector functions, potentially explaining the impaired anti-tumor response observed in HNSCC (47). In parallel, TNF-β and chemokines like CCL3 and CCL20 are critical for orchestrating immune cell recruitment and enhancing tumor surveillance. Their low expression may facilitate tumor immune escape and compromise local anti-tumor defense mechanisms (48). Conversely, we found elevated CCL2, IL-16, and IL-15 at one or both time points. These findings could represent compensatory immune or wound-healing responses.

We observed that levels of CCL2 in the peripheral blood of individual patients with HNSCC were significantly elevated post-operatively compared to healthy controls. This finding suggests the presence of an immunosuppressive environment within HNSCC, which may facilitate tumor progression and metastasis (49). The high levels of CCL2 are also in accordance with an increase in PD-L1^+^ myeloid tumor-infiltrating cells, indicating a potential mechanism through which HNSCC evades immune surveillance (50). The interplay between elevated CCL2 and PD-L1 expression suggests that the effectiveness of ICIs, such as atezolizumab and pembrolizumab, could vary significantly among patients, depending on their specific TME (51). Higher IL-15, meanwhile, has been associated with sustained NK cell and CD8^+^ T cell activity, suggesting that certain elements of the immune system remain activated or attempt to counterbalance immunosuppressive pressures (52). Elevated IL-16 prior to resection could reflect a tumor-driven inflammatory response, although the functional implications of this remain to be fully clarified. Interestingly, VEGFA and APRIL levels were not significantly altered, implying that these particular pathways may not be dominant drivers of the early postoperative immune environment in HNSCC. This observation complements studies indicating that the clinical impact of VEGF pathway perturbations can vary among patients, with some demonstrating robust changes while others show minimal shifts (53). However, a particularly noteworthy observation is that for many cytokines, the altered profiles detected prior to surgical resection remained largely unchanged 4–6 weeks post-operatively. This persistence of a dysregulated systemic cytokine environment, even after the removal of the primary tumor bulk, suggests several potential, non-mutually exclusive mechanisms. Firstly, it may indicate that the tumor has induced long-lasting systemic immunological alterations that are not immediately reversible upon tumor removal. This could involve lasting changes in immune cell populations, their functional status, or the establishment of chronic inflammatory states (54). Secondly, micrometastatic disease, not clinically apparent at the time of surgery, could continue to affect the systemic immune landscape (55, 56). Thirdly, the surgical intervention itself, despite its curative intent, can induce an inflammatory response and transient immunosuppression that might mask or delay the normalization of certain cytokine levels in the early postoperative period (57). Finally, it is also plausible that underlying patient-specific factors or comorbidities contribute to a baseline immune dysregulation that is exacerbated by the cancer but not exclusively dependent on the primary tumor’s presence. Future long-term follow up prospective clinical studies including patients treated with ICIs will help to identify most relevant expressed and secreted molecules to identify patterns, which are associated with ICI responses (58).

Tumor slices cultivation maintain a preserved structure and heterogeneity of the original tumor. The viability of tumor slices is retained for at least 72 hours but also cultivation periods for up to six or seven days have been reported to preserve viability and function. In some studies proliferating cells during this long-term cultivation, e.g., using breast cancer tissue (59), pancreatic ductal adenocarcinoma (15), colon cancer (60), human hepatocellular tumor (61), and different gastrointestinal malignancies (62) have been described. In this study, a cultivation period of six to seven days was chosen to facilitate the interaction of substituted autologous immune cells derived from peripheral blood. Precision-Cut Tumor Slices (PCTS) have been used for the investigation of cytotoxic treatment strategies e.g., in breast cancer to identify responders and non-responders (59). More recently, immune modulation was investigated also in HNSCC analyzing bispecific anti-CD3-EpCAM antibody or oncolytic virus activity (14). Different groups have used this technology to study checkpoint inhibitor strategies, for instance, Sivakumar et al. found increased frequency of CD3^+^ T cells and cytotoxic CD8^+^ T cell population in Hepatocellular carcinoma (HCC) tumor slices upon anti-PD-L1 treatment (61). Martin and colleagues identified potential responders to cytotoxic treatment and ICI (pembrolizumab) combination therapy in two out of nine metastatic colorectal cancer patients (63). The efficacy of novel checkpoint inhibitors were described by Jabbari, who identified cytotoxic treatment efficient synergistic killing with TIM-3 blockade (64). The inhibition of chemokine receptors (CXCR-4 inhibitor) in a pancreatic ductal adenocarcinoma revealed synergistic effect in combination with anti-PD-L1 treatment (65). In our investigations, atezolizumab showed a wide range of killing capacity and individual immune cell modulation whereas pembrolizumab was not able to induce effective killing. Comparison between α-PD-1 and α-PD-L1 efficiency have ben also investigated by Xing et al. who confirm our findings of individual responses in colon and breast cancer PCTS (60).

Voabil and colleagues also investigated PD-1 blockade efficiency in an *ex vivo* platform using NSCLC, breast, ovarian or renal cell carcinoma and found that patients’ samples with immune cell reactivation capacity predicted clinical response. This capacity was associated with the presence of tertiary lymphoid structures (13).

However, compared to the other studies described above, we added autologous immune cells from the peripheral blood of the patients to allow interaction with systemic effects of treatment in our culture system. This co-culture without matrigel or hydrogel enabled immune cell infiltration into the tumor tissue from supplemented autologous PBMCs. This presents an advantage over organoid culture systems that require scaffold material, which sometimes impede immune cell infiltration (66). Nevertheless, other investigators have demonstrated successful immune cell penetration in a scaffold based co-culture assay, summarized in (67).

An intriguing finding is that in some donor co-cultures atezolizumab (anti-PD-L1) caused more pronounced cell death than pembrolizumab (anti-PD-1) in this *ex vivo* setting. A number of factors might have an impact. First of all, PD-L1 is expressed not only on tumor cells but also on several immune cells, including B cells and especially myeloid cells within the TME (**Figure 1I**). Targeting PD-L1 with atezolizumab can thus block the interactions involving PD-L1 on tumor cells but also on immune with the effector cells. However, besides PD-L1 another ligand PD-L2 can interact with PD-1 expressing effector cells and can thereby inhibit their activity (68). Pembrolizumab targets PD-1 mainly expressed on T cells and other effector cells. However, the efficiency of both ICI varies when clinically applied but this phenomenon depends on the specific cancer type and treatment setting (69, 70). A direct comparative clinical trial between the two treatments has not been conducted. Moreover, receptor accessibility, receptor density, and immune cell infiltration affects the ICI response. These individual differences possibly influence treatment efficiency and 3D tissue assay may assist in the decision-making process.

Overall, PCTS allow the investigation of individual responses to ICI treatment in short-term intervals, which can serve as an element for personalized medicine in the future. However, large patient cohorts need to be prospectively evaluated for further validation. A direct statistical correlation of systemic soluble markers with the *ex vivo* functional readouts from the 3D co-cultures was not reasonable due to the limited number of fully paired patient samples across all assays. Nevertheless, correlation analyses done on larger cohorts are a reasonable perspective. Another limitation of PCTS technology is the possible inter-slice variations reflecting intra-tumor heterogeneity, which was compensated in this study by pooling multiple slices upon treatments. Other limitations are donor tissue availability and the restricted time-interval for cultivation. Finally, sufficient read-out parameters needs to be included, which allow the characterization of immune cell activation upon treatment. This includes detection of apoptosis induction in addition to the characterization of immune cell activation based in marker expression profiling and cytokine secretion assays.

## Data Availability

The original contributions presented in the study are included in the article/**Supplementary Material**, further inquiries can be directed to the corresponding author/s.
