# Supplementary figures and images for: Multiplexed immune profiling and 3D co-culture assays to assess the individual checkpoint therapy response in head and neck squamous cell carcinoma
Source: Front Oncol. 2025 Aug 8;15:1622008. doi: 10.3389/fonc.2025.1622008 (PMC12370455; doi:10.3389/fonc.2025.1622008)

*Supplementary Material*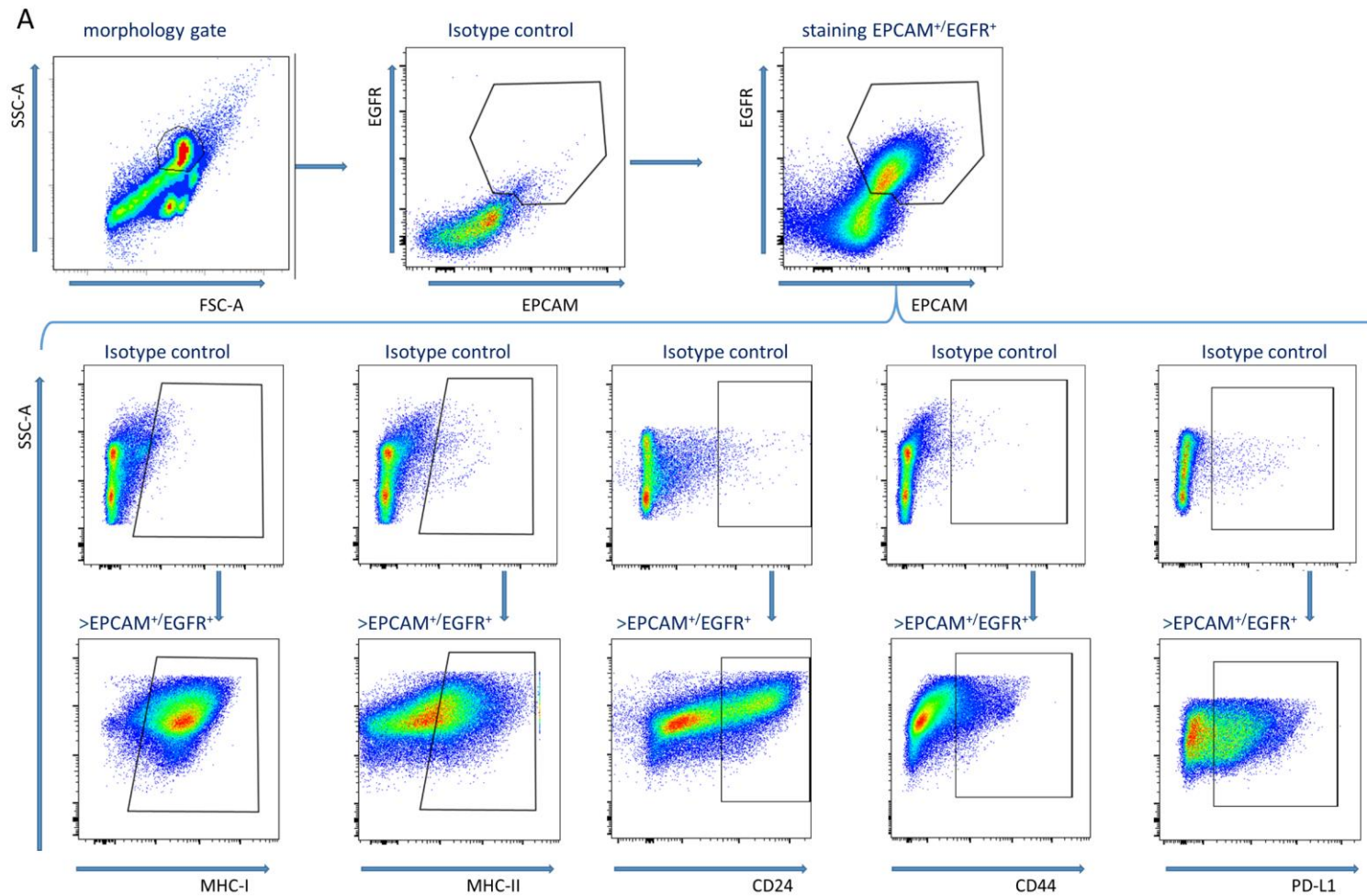

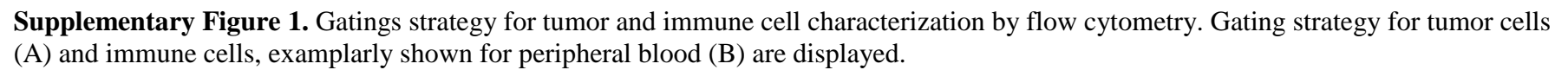

Supplement: Supplementary file 1 [file DataSheet1.pdf]
